# The Effect of Alcoholic Beverage Type on Hyperuricemia in Japanese Male Office Workers

**DOI:** 10.2188/jea.15.41

**Published:** 2005-05-10

**Authors:** Takuya Sugie, Takuya Imatou, Motonobu Miyazaki, Hiroshi Une

**Affiliations:** 1 Department of Epidemiology, National Institute of Public Health.; 2 Department of Hygiene and Preventive Medicine, School of Medicine, Fukuoka University.

**Keywords:** Alcohol, Alcoholic Beverages, Hyperuricemia, Uric Acid, office workers

## Abstract

BACKGROUND: The association between alcohol consumption and risk of hyperuricemia has been pointed out. However, the potential difference in the risk of hyperuricemia according to types of alcoholic beverage has not been assessed.

METHODS: A cross-sectional survey was performed using data from 715 men who had regular medical examinations in their workplace in 2001. Subjects were interviewed using a questionnaire about their lifestyle including types of alcoholic beverages and quantity of alcohol consumed per day. Logistic regression analysis was performed to assess the relationship between the types of alcoholic beverage and high serum uric acid level.

RESULTS: Compared with subjects who did not drink alcohol, the odds ratio (OR) of hyperuricemia (serum uric acid =7.0+ mg/dL) was 2.89 (95% confidence interval [CI]: 1.46-5.71) for subjects who consumed 50+ g/day of ethanol, and 2.64 (95% CI: 1.33-5.24) for subjects who consumed 25-49g/day. Compared with subjects who drank Japanese sake, subjects who drank beer (OR=1.24, 95% CI: 0.55-2.80) or shochu (OR=1.06, 95% CI: 0.44-2.51) did not have a statistically significant difference in risk for hyperuricemia.

CONCLUSION: These findings from a cross-sectional study of Japanese male office workers suggested that alcohol consumption is associated with an increased risk of hyperuricemia and that this increased risk does not vary according to the types of alcoholic beverage.

Many epidemiologic and biologic studies have suggested that serum uric acid is a major risk factor for gout.^1^^,^^2^ Besides gout, hyperuricemia has been associated with other diseases and conditions such as diabetes, hyperlipidemia, hypertension, and other cardiovascular diseases.^3^^-^^16^ In addition, some epidemiologic studies have suggested that hyperuricemia affects overall mortality, and that serum uric acid is a considerable risk factor for reduced life expectancy.^17^^,^^18^

Many previous studies have pointed out the risk factors for hyperuricemia.^8^^,^^9^^,^^19^^,^^20^ The association between alcohol consumption and hyperuricemia has also been suggested.^21^^-^^24^ In general, alcohol can increases serum uric acid levels associated with accelerated purine metabolism and decreased renal clearance of uric acid.^15^^,^^21^^,^^25^ Some studies suggested that the risk of hyperuricemia could vary depending on the type of alcoholic beverage,^24^^,^^26^^,^^27^ as does the risk of other diseases,^27^^,^^28^ but little data is available. Beer drinking in particular is considered to relate to hyperuricemia because it contains relatively high levels of purine,^24^ but the potential difference in the risk of hyperuricemia posed by different types of alcoholic beverage has not been thoroughly assessed.^24^ Whether there are differences in the risk of hyperuricemia among different types of alcoholic beverage is useful information with implications for the prevention and management of hyperuricemia. In order to examine these issues, we assessed the relationship between the types of alcoholic beverage consumed and risk of hyperuricemia.

## METHODS

### Study population

This survey was conducted among employees engaging in office work in Fukuoka, in the western part of Japan, in 2001. A total of 785 men among 826 employees over 35 years of age had annual health examinations. Men with high levels of serum creatinine, more than 1.4 mg/dL and under treatment for hyperuricemia, were excluded from this study. Based on chi-square analysis, significant associations were observed between serum uric acid level and alcohol intake, body mass index (BMI), eating snacks between meals, and smoking habits. If any of these four variables were unknown, the person was excluded. After these exclusions, 715 men participated in this study. Among the 715 subjects, 395 were regular drinkers of alcoholic beverages. In order to investigate the relationship between the alcoholic beverage type and high serum uric acid levels, 10 subjects were excluded who did not report the main alcoholic beverage consumed from beer, Japanese sake, which is an alcoholic beverage fermented from rice, or shochu, which is a traditional Japanese distilled spirit because they chose others. After all exclusions, 385 subjects were analyzed to assess the relationship between the alcoholic beverage types and high serum uric acid levels. Participation was voluntary and informed consent was obtained from each subject.

### Procedure

The survey included a physical examination, collection of blood samples for laboratory analysis, and a questionnaire. Subjects were interviewed about their lifestyle including alcoholic consumption using a questionnaire distributed in advance. Alcohol intake habit was examined in terms of type of alcoholic beverage and quantity of alcohol consumed per day. Subjects reported the frequency of alcoholic beverage consumption, which was used to group them into the following categories: non-drinkers (never or almost never drank alcohol in the past), occasional drinker (1 to 3 days per week), regular drinker (4 to 7 days per week). We calculated the unit measurement of ethanol for average daily intake of each beverage on the basis of known alcohol consumption. The average total and daily alcohol consumption were calculated assuming that 180mL (one unit) of Japanese sake, 633mL (one bottle) of beer, or 120mL (one unit) of shochu contains 25g of ethanol, which is the typical ethanol content of the Japanese sake, beer, and shochu consumed in Japan. Regular drinkers were classified into three categories according to the quantity consumed: less than 25g per day (<25 g/day), 25g to less than 50g per day (25-49 g/day), and 50g or more per day (50+ g/day). Regular drinkers were asked to choose the type of beverage that they usually consume from Japanese sake, beer, shochu and others. They were allowed to choose only one beverage. Other information collected by questionnaire included snack eating habits, smoking habits, exercise habits, and so on. Venous blood was taken for serum biochemical measurement after an overnight fast. Serum uric acid concentrations were determined with an auto analyzer (Hitachi 7350) by the uricase method.

### Statistical analysis

For statistical analysis, we took two steps. First, logistic regression analysis was performed to assess the relationship between alcohol intake and high serum uric acid level. Odds ratios were calculated from a multivariate models adjusted for age, BMI, intake of snacks, and smoking habits. In this analyses, among 715 subjects, cases were subjects whose serum uric acid levels were 7.0+ mg/dL (n=183) because hyperuricemia is defined as a serum uric acid level of 7.0 mg/dL or higher, without regard to sex or age, by the Japanese Society of Gout and Nucleic Acid Metabolism.^29^ Controls were subjects whose serum uric acid levels were less than 6.0 mg/dL (n=328) because the Japanese Society of Gout and Nucleic Acid Metabolism recommended to control the serum uric acid level less than 6.0mg/dL.^29^ Second, we investigated the relationships between the alcoholic beverage type and high serum uric acid levels. Logistic regression analysis was also performed to adjust the effects of age, alcohol intake, BMI, intake of snacks, and smoking habits. In these analyses, among 385 subjects who were regular drinkers and identified one main alcoholic beverage consumed, cases were subjects whose serum uric acid levels were also 7.0+ mg/dL (n=121) and controls were subjects whose serum uric acid levels were less than 6.0 mg/dL (n=148). All data were analyzed with SPSS^®^ Base 11.5J for Windows (SPSS Inc., Chicago, IL, USA).

## RESULTS

Table 1 shows the distribution of uric acid values by age group, alcohol intake habit, and BMI. Of the total population, 26% had serum uric acid levels of 7.0 mg/dL or higher and 9% of those had serum uric acid levels of 8.0 mg/dL or higher. Differences in the distribution of serum uric acid values were not significant among age groups. There was a significant difference in the distribution of uric acid level by alcohol intake habit. Table 2 shows odds ratios and 95% confidence intervals (CIs) for the risk of hyperuricemia by alcohol intake, adjusted for the effect of age, BMI, snack intake and smoking habits. Compared with subjects who did not drink alcohol, the OR was 2.89 (95% CI: 1.46-5.71) for subjects who consumed 50+ g/day of ethanol. The OR was 2.64 (95% CI: 1.33-5.24) for subjects who consumed 25-49 g/day. Table 3 shows characteristics of regular drinkers according to type of alcoholic beverage usually consumed. Table 4 shows the distribution of uric acid values by type of alcoholic beverage.

**Table 1.** Distribution of uric acid values by age, alcohol intake and body mass index.

	Serum uric acid level (mg/dL)				Total	

	<6.0	6.0-6.9	7.0-7.9	8.0+		
Age (years)						
35-39	61 (39)	45 (29)	33 (21)	16 (10)	155 (100)	
40-49	149 (46)	95 (30)	48 (15)	30 (9)	322 (100)	
50-59	103 (51)	55 (27)	31 (15)	14 (7)	203 (100)	
60+	15 (43)	9 (26)	8 (23)	3 (9)	35 (100)	p=0.558

Alcohol intake						
Non-drinkers	74 (56)	39 (29)	19 (14)	1 (1)	133 (100)	
Occasional drinkers	102 (55)	46 (25)	28 (15)	11 (6)	187 (100)	
Regular drinkers (daily ethanol consumption: g)						
<25	29 (45)	22 (34)	8 (12)	6 (9)	65 (100)	
25-49	60 (37)	53 (33)	32 (20)	17 (10)	162 (100)	
50+	63 (38)	44 (26)	33 (20)	28 (17)	168 (100)	p<0.001

Body mass index (kg/m^2^)						
<20.0	54 (67)	17 (21)	4 (5)	6 (7)	81 (100)	
20.0-21.9	49 (46)	43 (40)	13 (12)	2 (2)	107 (100)	
22.0-23.9	101 (54)	46 (24)	30 (16)	11 (6)	188 (100)	
24.0-25.9	67 (40)	52 (31)	35 (21)	15 (9)	169 (100)	
26.0+	57 (34)	46 (27)	38 (22)	29 (17)	170 (100)	p<0.001

Total	328 (46)	204 (29)	120 (17)	63 (9)	15 (100)	

Percentages in parentheses

**Table 2.** Odds ratios for hyperuricemia (7.0+ vs <6.0 mg/dL).

Alcohol intake	Cases(7.0+ mg/dL)	Controls(<6.0 mg/dL)	Odds ratio (95% CI)*	p
	
	No. (%)	No. (%)		
Non-drinkers	20 (11)	74 (23)	1.00 (reference)	
Occasional drinkers	39 (21)	102 (31)	1.20 (0.62-2.33)	0.59
Regular drinkers (daily ethanol consumption: g)				
<25	14 (8)	29 (9)	1.56 (0.66-3.71)	0.31
25-49	49 (27)	60 (18)	2.64 (1.33-5.24)	0.01
50+	61 (33)	63 (19)	2.89 (1.46-5.71)	0.00

* : Adjusted for the effect of age, body mass index, intake of snack, and smoking habits

CI: Confidence Interval

**Table 3.** Numbers of study participants according to type of alcoholic beverage usually consumed.

	Type of alcoholic beverage				Total

	Japanese Sake	Beer	Shochu	Others	
Age (years)					
35-39	12 (23)	56 (23)	9 (11)	2 (20)	79 (20)
40-49	20 (38)	122 (49)	37 (44)	7 (70)	186 (47)
50-59	18 (34)	65 (26)	30 (36)	1 (10)	114 (29)
60+	3 (6)	5 (2)	8 (10)	0	16 (4)

Alcohol intake (daily ethanol consumption: g)					
<25	11 (21)	35 (14)	15 (18)	4 (40)	65 (17)
25-49	25 (47)	97 (39)	35 (42)	5 (50)	162 (41)
50+	17 (32)	116 (47)	34 (41)	1 (10)	168 (43)

Body mass index (kg/m^2^)					
<20.0	2 (4)	36 (15)	2 (2)	0	40 (10)
20.0-21.9	4 (8)	48 (19)	13 (16)	2 (20)	67 (17)
22.0-23.9	16 (30)	71 (29)	22 (26)	2 (20)	111 (28)
24.0-25.9	12 (23)	49 (20)	23 (28)	4 (40)	88 (22)
26.0+	19 (36)	44 (18)	24 (29)	2 (20)	89 (23)

Snack between meals					
rare	3 (6)	8 (3)	4 (5)	1 (10)	16 (4)
sometimes	18 (34)	44 (18)	23 (27)	4 (40)	89 (23)
daily	32 (60)	196 (79)	57 (68)	5 (50)	290 (73)

Total	53 (100)	248 (100)	84 (100)	10 (100)	385 (100)

Percentages in parentheses

**Table 4.** Serum uric acid levels by type of alcoholic beverage.

Type of alcohol	Serum uric acid level (mg/dL)				Total

	<6.0	6.0-6.9	7.0-7.9	8.0+	
Japanese sake	20 (38)	17 (32)	8 (15)	8 (15)	53 (100)
Beer	91 (37)	80 (32)	48 (19)	29 (12)	248 (100)
Shochu	37 (44)	19 (23)	14 (17)	14 (17)	84 (100)

Total	148 (38)	116 (30)	70(18)	51 (13)	385 (100)

Percentages in parentheses

Among individual alcoholic beverages, the magnitude of the association for the risk of hyperuricemia was assessed by logistic regression analysis after adjusting for following other factors: Age, alcohol intake, BMI, snack intake, and smoking habits. Table 5 shows the ORs and 95% CIs for the risk of hyperuricemia according to the type of alcoholic beverages. Compared with subjects who drank Japanese sake, the OR was 1.24 (95% CI: 0.25-2.80) for subjects who drank beer, and 1.06 (95% CI: 0.44-2.51) for subjects who drank shochu. Compared with subjects who drank Japanese sake, subjects who drank beer or shochu did not have a statistically significant difference in risk for hyperuricemia.

**Table 5.** Odds ratios of types of alcoholic beverages for hyperuricemia (7.0+ vs <6.0 mg/dL).

Type of alcohol	Cases(7.0+ mg/dL)	Controls(<6.0 mg/dL)	Odds ratio (95% CI)*	p
	
	No. (%)	No. (%)		
Japanese sake	16 (13)	20 (14)	1.00 (reference)	
Beer	77 (64)	91 (62)	1.24 (0.55-2.80)	0.61
Shochu	28 (23)	37 (25)	1.06 (0.44-2.51)	0.90

* : Adjusted for the effect of age, body mass index, intake of snack, and smoking habits

CI: Confidence Interval

## DISCUSSION

We conducted a cross-sectional study to assess the association between alcoholic beverage type and serum uric acid level. In our results, although alcohol intake itself was associated with elevated serum uric acid, the type of alcoholic beverage was not found to be an independent risk factor for hyperuricemia. Compared with men who drank Japanese sake, men who drank beer or shochu did not have a significant difference in risk of hyperuricemia. Previous studies suggested an association between alcohol intake and hyperuricemia.^21^^,^^25^^,^^26^ There were also studies that assessed the difference in the risk of hyperuricemia for several alcoholic beverages. Kono et al. reported that among beer, Japanese sake, shochu and whisky consumption, only beer was significantly associated with an elevated level of serum uric acid level.^24^ Choi et al. also reported that alcohol intake is strongly associated with an increased risk of gout and that the risk varies substantially according to the type of alcoholic beverage: Beer confers a larger risk than spirits, whereas moderate wine drinking does not increase the risk.^26^ Regarding the different effects of alcoholic beverages on hyperuricemia, beer drinking is mentioned as a risk factor for hyperuricemia.^27^ Some previous studies suggested that certain non-alcoholic components that vary among alcoholic beverages play an important role in the incidence of hyperuricemia in metabolic mechanism. An example of a non alcoholic component is the variation in purine content among different alcoholic beverages.^27^^,^^30^ Beer is thought to be a risk factor for hyperuricemia because it is the only alcoholic beverage to have large purine content.^30^ On the other hand, our finding suggests a possibility that alcohol beverage itself, rather than the type of alcoholic beverage, is responsible for the risk of hyperuricemia. Other previous studies have demonstrated that alcohol can elevate serum uric acid levels through increased uric acid synthesis.^21^^,^^25^ Alcohol can also lead to hyperuricemia by decreased renal clearance of uric acid through alcohol induced elevation in blood lactate levels.^27^^,^^31^^,^^32^ We could not assess the metabolic mechanism by which alcohol itself elevate serum uric acid level without regard to the type of alcohol beverage. If alcohol itself is associated with hyperuricemia, this is useful knowledge for expanding a program for prevention of hyperuricemia. According to the National Nutrition Survey in Japan in 2002, 25.4% of the total population over 20 years of age has a habit of daily alcohol consumption. As for men older than 20 years of age, 49% of those drink alcoholic beverages daily.^33^ Excessive alcohol consumption is also related to stroke and cardiovascular diseases, although moderate alcohol intake might be able to reduce the risk of some diseases, including cardiovascular diseases.^34^^-^^38^ Our observations support generally reducing intake of alcoholic beverages to prevent hyperuricemia. Such findings will have some impact on the public health policy makers and consumers.

Besides gout, hyperuricemia is related to other diseases and conditions, such as diabetes, hyperlipidemia, hypertension, and cardiovascular diseases.^8^^-^^10^ More attention should be paid to increasing the awareness of hyperuricemia as a multiple risk factor for chronic diseases. On the other hand, there has been controversy as to whether hyperuricemia is an independent risk factor for cardiovascular disease development.^3^^,^^5^^,^^39^ Future studies should explain the metabolic mechanism of by which hyperuricemia is associated with cardiovascular diseases.^11^^-^^13^ Recently, an association between hyperuricemia and insulin resistance syndrome has been pointed out. Increased serum uric acid levels may be the result of an insulin-resistant state.^3^ This is supported by the finding that increased serum uric acid levels are related to decreased insulin-stimulated glucose uptake and increased plasma insulin response to oral glucose loading.^14^^-^^16^ It should be noted that preventing hyperuricemia by controlling alcohol consumption and controlling body weight could lead to preventing these diseases.

Although we did not observe a significant relationship between elevated serum uric acid level and alcoholic beverage type consumed, we recognize that we should estimate our results carefully because of some limitations. First, our investigation was based on a cross-sectional study. We could not assess the temporal relationship between the type of alcoholic beverage and the elevation of serum uric acid level. Second, information on alcohol consumption was obtained by an interview using a questionnaire without verification using biological markers. In addition, subjects reported only one beverage that they usually consumed. Many of the subjects might have consumed more than one kind of beverages. We could not examine clearly the effect of each single beverage, eliminating the effect of others. Considering these issues, although our results have to be interpreted with caution and further studies in assessing the effect of single alcoholic beverage will be expected, our findings suggest that alcohol intake itself rather than the type of alcoholic beverage should be focused on to prevent hyperuricemia. We assessed the effect on hyperuricemia of beer, Japanese sake, and shochu. These three are the most popular alcoholic beverages in Japan.^40^ Lifestyle in alcohol consumption in Japan is different from other countries. Wine, for example, still holds a fairly narrow share of the market.^40^ In the region where this study was conducted, people seemed to be distributed roughly into these three groups in terms of their preferred type of alcohol: beer, sake, and shochu. In this regard, this study is unique reflecting the alcohol intake habit in this region. Considering that this study was based on a cross-sectional design, a prospective study with a larger population will be expected to investigate the association between the type of alcoholic beverage and hyperuricemia. Studies of the metabolic mechanism how individual alcoholic beverages have effect on serum uric acid level will also be expected.

In conclusion, our findings from a cross-sectional study of Japanese male office workers suggested that alcohol consumption is associated with an increased risk of hyperuricemia and that this increased risk does not vary according to the type of alcoholic beverage.
